# Duplicated inferior vena cava-trifurcated portal vein: a rare anatomical variation encountered during Whipple procedure

**DOI:** 10.1093/jscr/rjad014

**Published:** 2023-01-26

**Authors:** Dimosthenis Chrysikos, Spiros Delis, Dimitra Smerdi, Eugenia Charitaki, Eirini Solia, Theodore Troupis

**Affiliations:** Department of Anatomy, Medical School, National and Kapodistrian University of Athens, Athens 15772, Greece; Department of General Surgery, Konstantopoulio General Hospital, Athens 14233, Greece; Department of Anatomy, Medical School, National and Kapodistrian University of Athens, Athens 15772, Greece; Department of General Surgery, Konstantopoulio General Hospital, Athens 14233, Greece; Department of Anatomy, Medical School, National and Kapodistrian University of Athens, Athens 15772, Greece; Department of Anatomy, Medical School, National and Kapodistrian University of Athens, Athens 15772, Greece

**Keywords:** duplicated inferior vena cava, anatomical variations, inferior vena cava, trifurcation portal vein

## Abstract

The inferior vena cava (IVC) is the largest single vein in humans. However, during embryogenesis, abnormalities can occur resulting in a duplicated IVC. The portal vein (PV) offers the main blood flood to the liver, forming by the left and right PV. A number of anatomical variations are noticed, underlying the great importance of the pre-operative imaging workup. This case report presents a duplicated IVC and a trifucated PV that were incidentally found in an 82 year-old Caucasian male with pancreatic ductal adenocarcinoma who underwent pancreatoduodenectomy (Whipple procedure). Although some anatomical variations, including the duplication of the IVC and the trifurcation of PV, may be rare to the general population, the suspicion of their existence should always be taken under consideration from surgeons during hepatobiliary or retroperitoneal operations.

## INTRODUCTION

The inferior vena cava (IVC) is the largest single vein draining the blood from the lower extremities, abdomen and pelvic area, whereas the portal vein (PV) is formed by the merge of splenic and superior mesenteric veins. Due to their complex embryological development, numerous anatomical variations have been reported [1–7]. Herein, we present a case of duplicated IVC and a trifurcated PV in a male patient, diagnosed with pancreatic ductal adenocarcinoma (PDAC). An embryological approach is included, as well as the most applicable classifications.

## CASE REPORT

**Figure 1:**
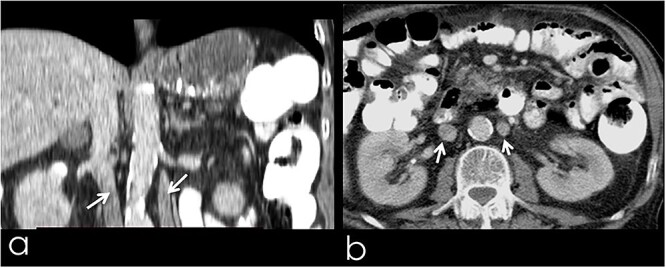
(**a**) Coronal image from CT with contrast, presenting the duplicated IVC (arrows); (**b**) transverse image from CT with contrast, presenting the duplicated IVC (arrows).

**Figure 2:**
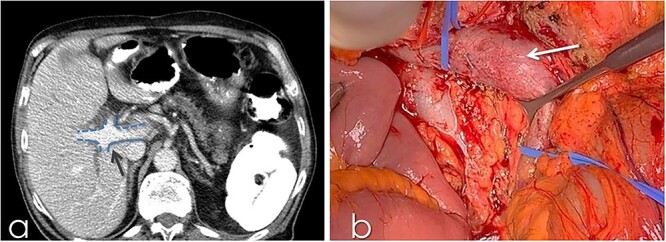
(**a**) CT with contrast demonstrating the trifurcated portal vein (arrow); (**b**) gross appearance of the duplicated IVC intraoperatively (arrow).

**Figure 3:**
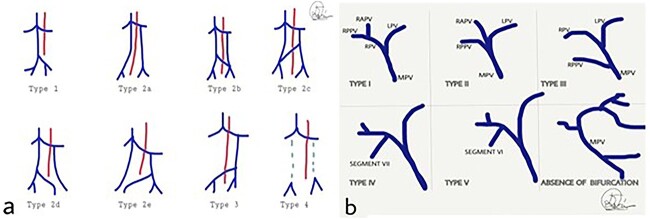
(**a**) Classification of IVC abnormalities; (**b**) variations of the branching of portal vein. MPV: main portal vein, RPV: right portal vein, LPV: left portal vein, RAPV: right anterior portal vein, RPPV; right posterior portal vein.

An 82-year-old Caucasian male was diagnosed with obstructive jaundice due to pancreatic duct adenocarcinoma. Computer tomography (CT) and magnetic resonance imaging (Figs 1 and 2) revealed a resectable tumor of the head of the pancreas, a duplicated IVC and a PV trifurcation. The two common iliac veins did not join together at lumbar 4 (L4) vertebra level but ascended separately along the celiac aorta, representing right and left IVC. Specifically, the right external and internal iliac veins joined, forming the right common iliac vein that ran along the abdominal aorta, representing the right IVC. The left IVC formed exactly like the right one while draining to the left renal vein (Fig. 1). This variation can be characterised as ‘type 2a’ in accordance with the classification shown in Fig. 3 (type 2a). Moreover, in our patient, a Whipple procedure was performed without post-operative complications.

Duplication of the IVC is a rare anatomical entity that did not affect the surgical treatment in our case per se. However, in cases of borderline or locally advanced pancreatic adenocarcinomas, duplicated IVC should be taken under consideration, as the left renal vein cannot be used as a conduit for PV replacement. Another interesting aspect refers to the right IVC invasion in case of PDAC and double IVC; in this case, the duplication may help the hepatobiliary surgeon sacrifice the main vessels due to multiple retroperitoneal collaterals. Thus, this hypothesis needs to be proven anatomically.

Another important aspect in locally advanced PDAC is the portal-mesenteric complex replacement, followed by the performed splenorenal shunt. Anatomy knowledge is of great importance in such circumstances, as it may affect the technique of reconstruction.

## DISCUSSION

### Duplication of IVC

The duplicated IVC appears with an incidence of 0.2–3%. [2, 8] A suggested classification is shown in Fig. 3 [9] Type 1 refers to normal IVC formation of the common iliac veins connection. Type 2a represents a duplicated IVC without communication between the two common iliac veins, whereas types 2b and 2c refer to duplicated IVC with interiliac communication from the left and right common iliac veins, respectively. Additionally, type 2d and 2e stand for duplicated IVC with interiliac communication from left and right internal iliac veins, respectively. In type 3, the left IVC formed by the merge of left and right common iliac veins. In type 4, there is no iliac connection and the infrarenal IVC is absent. In our case, the duplicated IVC is characterised as type 2a [9].

In the fourth week of embryogenesis, three venous systems are formed: the vitelline, the umbilical and the cardinal system. The IVC consists of four main segments: [2, 5] the hepatic segment derives from the hepatic vein and the hepatic sinusoids; the prerenal segment originates from the right subcardinal vein; the renal segment is derived from the anastomosis of the right subcardinal anteriorly and supracardinal vein posteriorly; and the last one, the postrenal system, arises from the right supracardinal vein. Normally, the left subcardinal and supracardinal veins regress. In rare cases, the veins do not regress, leading to duplication, the persistence or the combination of both conditions. [8]

Most cases of duplicated IVC are asymptomatic. Its recognition is important intraoperatively, as it can prevent several complications such as hemorrhage, during interventional vascular practice, and prevent the misconception of the duplicated vessel as lymph nodes or masses. [2, 10] Furthermore, this variation is related to about 5% of the deep vein thrombosis (DVT) and should be suspected in the recurrence of pulmonary thromboembolism. There is a debate concerning if the duplicated IVC is associated with an increased possibility of DVT [8]. In a case report, [11] an 18-year-old woman was diagnosed with a DVT and a duplicated IVC, raising awareness for the relation between these two entities.

## TRIFURCATION OF PV

The PV is formed by the vitelline venous system. In the fifth week of embryogenesis, the left and right vitelline veins form a mesh that surrounds the duodenum through a group of ventral and a single dorsal communicatory venous component. At 10 weeks’ gestational age, the main portal vein (MPV) is formed by the left vitelline vein and the dorsal component, the left portal vein (LPV) arises from the left vitelline vein and ventral anastomoses and the right portal vein (RPV) emerges from the right vitelline vein. Deviations from this pattern lead to numerous anatomical variants [6, 12, 13].

Variations of PV anatomy are encountered in 20–35% of the population [6, 14], and their knowledge is crucial in PV embolisation, liver resection, liver transplantation, etc., minimising the post-operative mortality and morbidity [7].

Figure 3 presents the existing classification. [7, 13, 15] Type I shows the normal PV formation. In type II, the trifurcation of the PV is presented, whereas in type III, the right posterior PV (RPPV) represents the first branch of the MPV. Type IV and V refer to the separate origin of the branches of segments VII and VI correspondingly from the MPV. The last variant mentions the absence of PV bifurcation. In our case, a trifurcated PV, type II according to this classification, was noticed.

To conclude, deep knowledge of typical vascular anatomy and anatomical variations is essential as surgeons should be prepared and confident to perform operations safely and effectively, in order to avoid inadvertent complications that may be fatal.

## CONSENT FORM

Written and signed consent form has been received from the patient/family member and will be able to be reproduced upon request.

## AUTHORS’ CONTRIBUTIONS

D.C., S.D.: conceptualisation; D.S., D.C., E.S., manuscript writing, editing and final revision; S.D., D.S., E.C., E.S.: data collection; D.S.: visualisation and technical support; T.T.: supervision. All authors approved the final draft of the manuscript.

## DATA AVAILABILITY

The data that support the findings of this case report are available from the corresponding author [DC] upon reasonable request and in accordance with Consent Form.

## CONFLICT OF INTEREST STATEMENT

None declared.

## FUNDING

The authors declare no funding.
